# Frailty Test Battery Development including Physical, Socio-Psychological and Cognitive Domains for Cardiovascular Disease Patients: A Preliminary Study

**DOI:** 10.3390/jcm11071926

**Published:** 2022-03-30

**Authors:** Nastasia Marinus, Carlo Vigorito, Francesco Giallauria, Paul Dendale, Raf Meesen, Kevin Bokken, Laura Haenen, Thomas Jansegers, Yenthe Vandenheuvel, Martijn Scherrenberg, Joke Spildooren, Dominique Hansen

**Affiliations:** 1 REVAL-Rehabilitation Research Center, Faculty of Rehabilitation Sciences, Hasselt University, 3590 Diepenbeek, Belgium; raf.meesen@uhasselt.be (R.M.); kevin.bokken@student.uhasselt.be (K.B.); laura.haenen@student.uhasselt.be (L.H.); thomas.jansegers@student.uhasselt.be (T.J.); yenthe.vandenheuvel@student.uhasselt.be (Y.V.); joke.spildooren@uhasselt.be (J.S.); dominique.hansen@uhasselt.be (D.H.); 2 BIOMED-Biomedical Research Center, Hasselt University, 3590 Diepenbeek, Belgium; paul.dendale@jessazh.be (P.D.); martijn.scherrenberg@jessazh.be (M.S.); 3 Department of Translational Medical Sciences, Federico II University of Naples, 80131 Naples, Italy; vigorito@unina.it (C.V.); francesco.giallauria@unina.it (F.G.); 4 Faculty of Science and Technology, University of New England, Armidale, NSW 2350, Australia; 5 Heart Centre Hasselt, Jessa Hospital, 3500 Hasselt, Belgium

**Keywords:** frailty, frailty assessment, cardiovascular disease, older adults

## Abstract

Frailty is an age-related decline in physical, socio-psychological and cognitive function that results in extreme vulnerability to stressors. Therefore, this study aimed to elucidate which tests have to be selected to detect frailty in a comprehensive and feasible manner in cardiovascular disease (CVD) patients based on multivariate regression and sensitivity/specificity analyses. Patients (*n* = 133, mean age 78 ± 7 years) hospitalised for coronary revascularisation or heart failure (HF) were examined using the Fried and Vigorito criteria, together with some additional measurements. Moreover, to examine the association of frailty with 6-month clinical outcomes, hospitalisations and mortality up to 6 months after the initial hospital admission were examined. Some level of frailty was detected in 44% of the patients according to the Vigorito criteria and in 65% of the patients according to the Fried criteria. Frailty could best be detected by a score based on: sex, Mini Nutritional Assessment (MNA), Katz scale, timed up-and-go test (TUG), handgrip strength, Mini-Mental State Examination (MMSE), Geriatric Depression Scale (GDS-15) and total number of medications. Frailty and specific markers of frailty were significantly associated with mortality and six-month hospitalisations. We thus can conclude that, in patients with CVD, sex, MNA, Katz scale, TUG, handgrip strength, MMSE, GDS-15 and total number of medications play a key role in detecting frailty, assessed by a new time- and cost-efficient test battery.

## 1. Introduction

Almost half of all (premature) deaths in Europe are caused by cardiovascular diseases (CVDs). As about 10% of Europeans currently suffer from CVD, a significant economic cost and burden are apparent [1,2,3]. Moreover, due to increasing prevalence rates of obesity, hypertension and diabetes mellitus, a 10% increase in the CVD prevalence rate is expected in the upcoming 10 years [4].

Fortunately, improvements in cardiac surgery [5] and rehabilitation [6,7,8], risk factor management [9] and cardioprotective medication [2] have considerably increased the life expectancy of CVD patients [2,9]. However, ageing is commonly associated with the emergence of frailty [8]. Frailty is a progressive age-related decline in physiological systems that results in decreased reserves of intrinsic capacity, which confers extreme vulnerability to stressors [10]. This condition further increases the risk of adverse health outcomes, such as frequent hospitalisations and premature death, and therefore deserves great attention [1,11]. 

The prevalence rates of frailty in CVD patients can vary significantly according to the disease and treatment: from up to 19% in patients after percutaneous coronary intervention (PCI) to up to 76% in heart failure (HF) patients [12]. In these studies, the phenotype proposed by Fried [13] was the most frequently used frailty assessment tool. As mainly physical limitations are taken into account in this tool (i.e., weight loss, physical activity, walk time and handgrip strength), previous studies highlighted the need for a more comprehensive frailty assessment for better prediction of clinical outcomes in hospitalised older (CVD) patients [14,15,16]. 

For example, postoperative cognitive dysfunction (POCD), defined as the development of symptoms of cognitive dysfunction after surgery and anaesthesia in previously apparently cognitive healthy patients [17], occurs after cardiothoracic surgery in up to 43% of older patients [18,19,20] and can become a permanent disorder [21,22]. Moreover, depression (eventually in combination with anxiety), as well as a lack of social/emotional support in CVD patients, seems to be associated with adverse cardiovascular outcomes and mortality in a dose–response relationship [23]. Consequently, it is clear that besides the physical aspects of frailty, equal attention should be directed to the cognitive, social and psychological components of frailty as well, as already reiterated by the European Association of Preventive Cardiology [24,25] and more recently in the frailty score proposed by Vigorito [26]. In contrast to Fried et al. [13], this multidimensional frailty assessment tool takes into account not only the physical aspects of frailty (muscle strength, gait speed, mobility, comorbidities) but also nutritional, cognitive and psychosocial components with separate cut-off criteria for men vs. women. However, this Vigorito frailty assessment tool is not yet validated in CVD patients. 

Therefore, if the Fried and Vigorito criteria and some other frequently used frailty assessment measurements were to be merged, the tests that should be selected to establish a comprehensive assessment that is feasible and low cost but sufficiently sensitive and specific (females vs. males) remain to be determined [12]. Such an assessment battery would then allow clinicians, working in different settings, to easily detect frailty and, moreover, predict hospitalisations and mortality in patients with CVD to initiate preventive strategies accordingly. 

The aim of this study, therefore, was threefold: (1) to compare the frailty prevalence rates using Fried vs. the more comprehensive Vigorito criteria in CVD patients; (2) to establish which tests, from the physical, socio-psychological and cognitive domains, should be selected to be able to detect frailty in patients with CVD and (3) to establish a total score that may represent a valid measurement of frailty severity. 

## 2. Materials and Methods

### 2.1. Subjects

Between October 2019 and April 2020, 133 unselected, consecutive participants were included in this cross-sectional study at the cardiology units of Jessa Hospital Hasselt, Belgium. Hospitalised participants were initially screened for inclusion and exclusion criteria based on their electronic patient file and, if necessary, based on additional information from the health staff (cardiologists, nurses) of the cardiology units of the hospital. After careful explanation of the study aims and methodology, written informed consent was obtained from all participants. This study was approved by the ethical committee of Jessa Hospital (19.81-REVA19.05) and registered at ClinicalTrials.gov (NCT04206904). The inclusion criteria were (i) men and women aged 65 years or older (ii) who were admitted to the hospital for mild vs. severe coronary revascularisation or surgery (PCI vs. (endo-)CABG) or for HF. We preferred to include these different CVD pathologies based on previous literature confirming the variable frailty prevalence in these patient populations [12]. Participants were excluded if they refused to participate after receiving all study information or if they had a persistently unstable clinical condition that prevented them from safely participating, such as angina pectoris, advanced conduction disturbances, significant ventricular arrhythmias or decompensating HF. Participants were not excluded based on mental/cognitive state. 

### 2.2. Study Design

In this cross-sectional study, the presence of frailty was initially assessed by two different frailty assessment tools. First, the presence/absence of frailty was examined according to the phenotype proposed by Fried [13]. Next, this frailty assessment was supplemented by the comprehensive multi-component and sex-specific frailty assessment tool proposed by Vigorito et al. [26], which was developed based on similar, previously published frailty assessment tools [14,15,16]. Furthermore, additional parameters were assessed, which could be of significant added value in the detection of frailty. The total test battery took 45 min to complete. 

Patients undergoing coronarography, further defined as PCI patients (for coronary artery disease (CAD) patients undergoing a PCI) or as CORO patients (for CAD patients not undergoing PCI or CABG), were examined before or after their cardiac surgery, while CABG patients were all examined before surgery. HF patients were examined at any defined time during their hospital stay. 

### 2.3. Baseline Characteristics 

Baseline characteristics (age, body weight and length) were registered from the electronic file of the patients on the day of assessment. 

### 2.4. Frailty Assessment 

#### 2.4.1. Fried Phenotype

The Fried frailty phenotype examines five components: involuntary weight loss, exhaustion, level of physical activity, walking time and grip strength. Based on these five criteria, subjects were considered to be pre-frail (fulfilling one or two criteria) or frail (fulfilling at least three criteria). A more detailed explanation of the different components can be found in Appendix A Table A1. 

With regard to the walking time criteria, the walking time of the slowest participant was assigned to participants who were not able to execute the walking test due to, for example, walking difficulties or exhaustion. In this way, we were able to calculate a mean walking time for the total sample. 

Furthermore, the Minnesota Leisure Time Activity questionnaire, which is used in the original Fried criteria, is largely inapplicable to hospitalised patients, as it examines participation in daily activities such as mowing the lawn, gardening, biking, dancing, swimming, etc. Therefore, we decided to use a modified version of the Fried phenotype by introducing the Katz scale. This scale has been used in previous studies to examine the level of physical activity according to the Fried phenotype [12,27,28,29]. It examines participation and level of (in)dependence in six activities (washing, dressing, mobility, toileting, level of (in)continence and eating) that are highly relevant for hospitalised patients. Based on this scale, subjects who were completely independent in 6 activities of daily living (ADL) (score 6: 1 point for each activity in which there was complete independence) were considered to be non-frail, while subjects with any dependence (score 0–5) were considered to be frail with regard to the level of physical activity. 

#### 2.4.2. Vigorito’s Frailty Assessment Tool 

The frailty assessment tool developed by Vigorito et al. [26] is composed of eight main components. 

The Mini Nutritional Assessment (MNA) (long version) [30] was used to examine the nutritional status of the patient. To examine the level of (in)dependence in activities of daily living (ADL), the Katz scale was used. Mobility was evaluated by measuring the gait speed based on a 4.6 m walking test. A combination of mobility, balance and lower-extremity strength was assessed based on the timed up-and-go test (TUG). To be able to calculate the mean gait speed or TUG score for the total sample, the value of the slowest participant (i.e., lowest value for gait speed or highest value for TUG) of the total sample was assigned to participants who were not able to execute the mobility tests due to, for example, walking difficulties or exhaustion. 

Handgrip strength (kg) of the dominant hand was examined with the Jamar handheld dynamometer^®^ (Patterson Medical, Glossop, UK) [31]. However, when the dominant hand was medically unfeasible due to, for example, a PCI/stenting procedure on that hand, the non-dominant hand was tested. Moreover, to be able to calculate the mean handgrip strength of the total sample, the value of the weakest participant (i.e., lowest value) of the total sample was assigned to participants who were not able to squeeze with any hand due to, for example, exhaustion. 

The Mini-Mental State Examination (MMSE) (Dutch version) [32] was used to examine the cognitive status of the patients. To detect the presence of a depressive mood, the Geriatric Depression Scale (GDS-15) (Dutch version) [33] was used. Finally, the use of cardioprotective and any other medications (except for vitamins, minerals and food supplements) was registered as a marker of comorbidities based on the electronic file of the patient at discharge from the hospital. Each component of the frailty assessment tool was scored separately to divide the patients into three frailty categories from not frail (score 0) to severe frailty (score 3). These eight sub-scores finally resulted in a total score ranging from not frail (score 0–6), minor frailty (7–12) and moderate frailty (score 13–18) to severe frailty (score 19–24) (see Appendix A Table A2). 

#### 2.4.3. Additional Frailty Measures 

In addition to both frailty assessment tools, other measurements were executed to collect extra information regarding the functional status of the patient in an attempt to improve frailty assessment.

The International Physical Activity Questionnaire (IPAQ) [34] (long version) was used to examine the level of physical activity spent in the previous seven days. To examine the muscle strength (in kg) of the knee extensors (sitting position with hip and knee flexed 90°) and hip flexors (supine position with hip flexed 90°) of both legs, the MicroFET^®^ dynamometer (Hoggan Health Industries Inc., West Jordan, UT, USA) [35] was used. Each measurement was repeated three times, and the highest value was used in the data analysis. Moreover, to examine the functional muscle strength of the lower limbs, the timed chair stand test was performed. The value of the weakest participant (lowest value (Microfet) and highest value (timed chair stand test)) of the total sample was assigned to participants who were not able to perform the muscle strength measurements due to, for example, exhaustion. Finally, the Falls Efficacy Scale International (FES-I) [36] was used, a questionnaire that examines the level of concern about falling (see Appendix A Table A3). 

All frailty assessment tools were implemented by trained physiotherapists. The data analysis was performed by another blinded researcher. 

### 2.5. Association of Frailty with 6-Month Clinical Outcomes

To examine the association of frailty with clinical outcomes, six months after the hospital admission in which the initial frailty assessment took place, the presence/absence of hospitalisations and mortality were examined based on records in the electronic patient file. A distinction was made between planned and urgent hospitalisations. Planned hospitalisations were considered to be hospital admissions that were planned in advance, such as a planned coronarography, PCI or valve surgery. Urgent hospitalisations were considered hospital admissions that were not planned in advance, such as hospitalisations via the emergency department of the hospital. 

Patients were considered to be frail when fulfilling at least three out of five criteria (Fried) indicating the presence of mild, moderate or severe frailty (Vigorito) or based on the newly developed frailty cut-off score (new frailty assessment tool) (further explained in detail in Section 3.5). 

### 2.6. Outcome Measures

The primary outcomes of this study were the frailty score and frailty characteristics based on the comprehensive frailty assessment battery developed by Vigorito (and additionally, according to the Fried phenotype). Secondary outcomes were hospitalisations and mortality 6 months after the initial frailty assessment. 

### 2.7. Statistical Analysis 

Statistical analyses were executed in SPSS v. 25.0 (IBM, Chicago, IL, USA) and JMP^®^ Pro 14.1.0 (SAS Institute Inc., Buckinghamshire, UK). Shapiro–Wilk tests were used to test for normality, while Levene’s tests for equality of variances were used to test for homoscedasticity. To compare two means, an independent samples *t*-test (in the case of normality) or a non-parametric Mann–Whitney U test (in the case of non-normally distributed data or sample size < 30) was used. Pearson chi-square or Fisher exact test (if cell number < 5) was performed to examine categorical data. To compare more than two means, one-way ANOVA (with Bonferroni test) (in the case of normality) or Kruskal–Wallis test (with pairwise comparisons) (in the case of non-normally distributed data) was used. A stepwise multivariate regression model was used in JMP to examine which specific components of frailty (age, sex, body length, body weight, BMI, MNA, calf circumference and upper arm circumference (which are part of the MNA), Katz scale, walking time, gait speed, TUG, handgrip strength, FES-I, MMSE, GDS-15, number of medications, muscle strength of knee extensors and hip flexors (left/right leg), timed chair stand test, CVD risk factors (hypertension, hypercholesterolemia, diabetes type 1, diabetes type 2, smoking), total number of risk factors and IPAQ) would predict the total frailty score the best and to develop a frailty assessment tool with the fewest assessments. In the case of correlating variables, such as gait speed and walking time, only one of the two variables was included in the analysis. To examine the association of frailty with 6-month clinical outcomes, chi-square analyses were performed between the presence/absence of planned/urgent hospitalisations or mortality and the frailty status of the patients (frail/not frail) according to Fried or Vigorito. Data are expressed as means ± standard deviation (SD) or as *n* (%). A *p*-value < 0.05 (2-tailed) was considered as statistically significant. 

## 3. Results

### 3.1. Baseline Characteristics 

This study included 133 participants (57 females) with a mean age of 78 ± 7 years, comprising 27 CORO patients, 30 PCI patients, 16 CABG patients and 60 HF patients. HF patients were significantly older compared to CORO (*p* = 0.002) and PCI patients (*p* = 0.002) (see Table 1).

### 3.2. Prevalence of Frailty According to the Fried Phenotype

**Figure 1:**
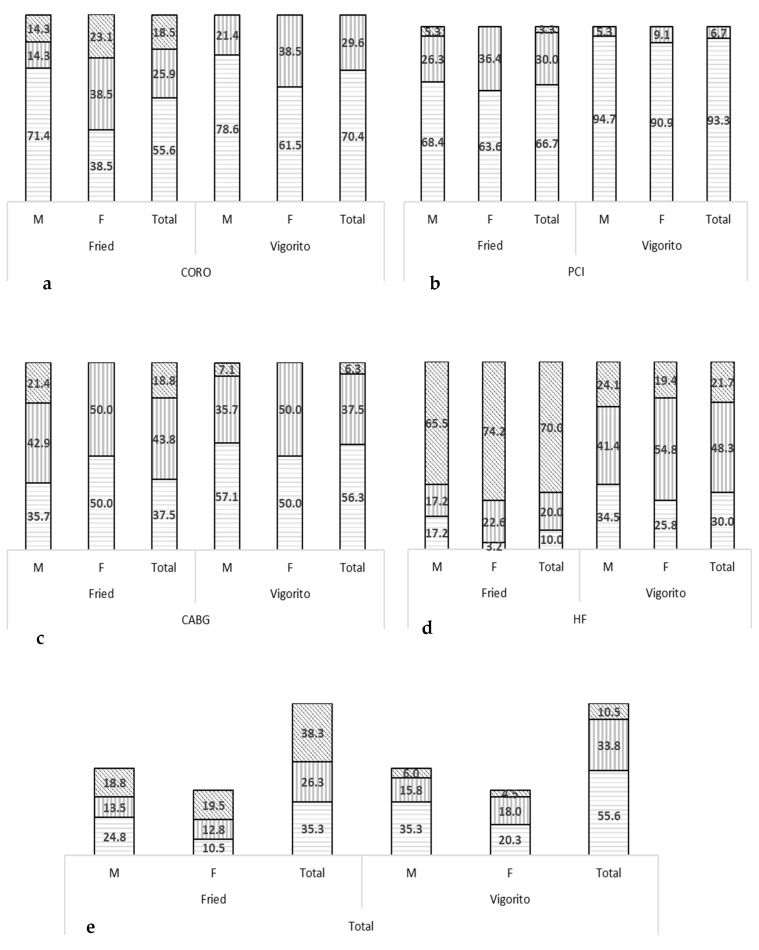
Distribution of the different levels of frailty (%) according to Fried and Vigorito for male and female CORO (**a**), PCI (**b**), CABG (**c**) and HF (**d**) patients. The different levels of frailty are represented as no frailty (horizontal lines), pre-frailty (Fried) or mild frailty (Vigorito) (vertical lines) and frailty (Fried) or moderate frailty (Vigorito) (diagonal lines). Note: Severe frailty (Vigorito) was not detected in the subjects and, thus, are not represented in the figure. Results are expressed as % within males and within females per CVD for each subcategory of frailty (in CORO, PCI, CABG and HF patients) or as % within CVD for total results (in CORO, PCI, CABG and HF patients) or as % within total population (for total results in last graph) (**e**). CABG, coronary artery bypass grafting; CORO, coronarography; F, females; HF, heart failure; M, males; PCI, percutaneous coronary intervention.

According to the Fried phenotype, 38% of the patients were categorised as being frail, while 26% of the patients were pre-frail (no significant difference, *p* = 0.08). The highest prevalence of frailty was detected in the HF patients (70%), with lower prevalence rates in CABG (19%), CORO (19%) and PCI (3%) patients. Major differences between HF patients and other patient populations were identified for nearly all outcomes (*p* < 0.05). Moreover, frailty was more prevalent in females than in males in the total population (46% vs. 33% respectively) and within each CVD individually because of significant differences in gait speed, handgrip strength and exhaustion (*p* < 0.05) (see Table 2 and Figure 1).

### 3.3. Frailty Characteristics Based on Vigorito et al.’s Frailty Assessment Tool

Based on the comprehensive multi-perspective frailty assessment tool developed by Vigorito et al. [26], 44% of the patients were categorised as having minor to severe frailty, of which significantly more CVD patients suffered from minor vs. moderate frailty (34% vs. 10%, *p* < 0.001), while severe frailty was not detected. The highest prevalence of frailty was detected in HF patients (70%) and CABG patients (44%), while the frailty prevalence rates were lower in CORO (30%) and PCI (7%) patients. Major differences between HF patients and other patient populations were identified for nearly all outcomes (*p* < 0.05). Moreover, frailty was more prevalent in females than in males (53% vs. 38%, respectively) in the total population and within each CVD individually because of significant differences in gait speed, handgrip strength and TUG (*p* < 0.05) (see Table 3 and Table 4 and Figure 1).

### 3.4. Comparison between Vigorito and Fried Frailty Criteria

Some level of frailty was detected in 44% of the patients according to Vigorito et al.’s frailty assessment tool (from mild to severe frailty) and in 65% of the patients according to the Fried phenotype (from pre-frail to frail) (x^2^ = 57.95, *p* < 0.001) (see Figure 1). However, according to Vigorito et al.’s tool, significantly more CVD patients suffered from minor vs. moderate frailty (34% vs. 10%, *p* < 0.001), while the Fried phenotype did not succeed in detecting any significant difference in the number of pre-frail vs. frail patients (26% vs. 38%, *p* = 0.11). 

Moreover, 51 patients were detected as being frail according to Fried. However, of these patients, Vigorito criteria classified 25% as having moderate frailty, 69% as having minor frailty and 6% as being non-frail. Similarly, of the 35 patients classified as pre-frail according to Fried, only 3% of the patients were classified as having moderate frailty, and 29% had minor frailty, while 69% of them were not frail according to Vigorito. As the largest proportion of pre-frail patients based on Fried seem to not be frail according to Vigorito and frail patients based on Fried seem to mainly have minor frailty according to Vigorito, we suggest that, based on these data, the Fried criteria may overestimate frailty and its severity. The same findings emerged when a comparison was made between older and younger CVD patients. Moreover, based on this analysis, a significant association was found between age and frailty status (see Appendix A Table A4). 

### 3.5. Creation of New Frailty Test Battery 

To examine which frailty measurements could contribute to the prediction of frailty in CVD patients and should thus be executed in clinical settings, multivariate correlations between all frailty assessments (in particular, the components of the Fried and Vigorito frailty assessments and all additional frailty measurements) and the total frailty score according to Vigorito et al. were determined. From these analyses, the following parameters correlated significantly (*p* < 0.05) with the total Vigorito frailty score: walking time (r = 0.854), TUG (r = 0.845), gait speed (r = −0.823), TCST (r = 0.740), MNA (r = −0.727), Katz scale (r = −0.694), number of medications (r = 0.641), handgrip strength (r = −0.607), MMSE (r = −0.559), knee extension strength (right leg) (r = −0.549), hip flexion strength (right leg) (r = −0.548), hip flexion strength (left leg) (r = −0.539), GDS−15 (r = 0.531) and knee extension strength (left leg) (−0.526). 

Finally, a multivariate regression model was built to decide which test should be maintained so that it has as few measurements as possible but optimal predictive power. In this model, the total frailty score of Vigorito et al.’s frailty assessment tool was considered the dependent variable, while all frailty assessments/parameters were considered independent variables. To detect frailty (R^2^ = 0.95), sex, MNA, Katz scale, TUG, handgrip strength, MMSE, GDS-15, total number of medications and the interaction of Katz scale and TUG should be assessed. 

Based on these parameters, which are components of Vigorito et al.’s frailty assessment tool, a new formula was developed (r = 0.98 with Vigorito score, *p* < 0.001):

Total frailty score = [(18.221173 + (1.1454217 × sex] + (−0.267283 × MNA score)] + (−0.947011 × Katz scale score) + (0.2157993 × TUG score) + (−0.081659 × handgrip strength score) + [−0.18281 × MMSE score) + (0.2700342 × GDS-15 score) + (0.2264091 × total number of medications) + [0.0453303 × (Katz scale score − 5.21805) × (TUG score − 14.3608)]]

**Figure 2:**
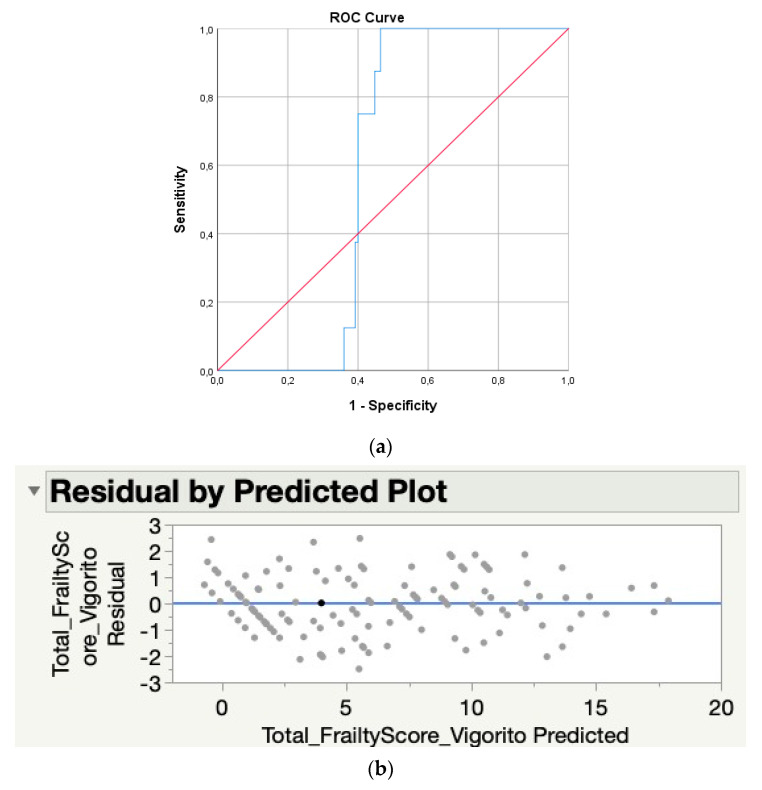
ROC curve (**a**) and plot (**b**) of the new regression formula vs. the total frailty score of Vigorito et al.

In order to avoid false-negative frailty diagnoses, a sensitivity of 1.0 was determined with a corresponding specificity of 0.54, resulting in a cut-off score of ≥5.56 pointing towards frailty according to this newly proposed frailty score (see Table 5 and Figure 2). 

### 3.6. Association of Frailty with 6-Month Clinical Outcomes

To examine the association of frailty with clinical outcomes, hospitalisations and mortality up to six months after the initial hospital admission were examined. During this period, 39% of the patients were readmitted to the hospital, and 56% of these hospitalisations were attributed to HF patients. The hospital admissions were mainly of cardiovascular, pulmonary or metabolic origin (65%), followed by orthopaedic (e.g., falls, fractures, amputations) (13%) and neurological events (e.g., stroke) (3%), while 20% were classified as another event (e.g., epistaxis, wound problems, hematomas, etc.). Six months after the initial hospital admission, 7% of the subjects died, of which 89% were HF patients (OR 11.1). 

Significant associations between (markers of) frailty and 6-month clinical outcomes can be found in Table 6. Frailty and specific markers of frailty (e.g., handgrip strength) were significantly associated with mortality and 6-month general, urgent, orthopaedic and cardiovascular hospitalisations. Especially orthopaedic hospital admissions were associated with frailty and several frailty components. Furthermore, specific Vigorito components are more feasible for predicting mortality, while specific Fried components can better predict 6-month (urgent) hospitalisations.

Moreover, subgroup analysis (HF vs. CAD) did not reveal any significant associations with the newly proposed frailty assessment battery. 

## 4. Discussion

This was the first study that aimed to analyse the prevalence of frailty in hospitalised CVD patients using the Fried vs. Vigorito criteria. Moreover, we were able to define which tests should be included in such an assessment to generate a time- and cost-efficient frailty assessment tool for CVD patients, allowing the development of a multi-component and sex-specific frailty assessment tool. 

In this study, 70% of HF patients and 44% of CABG patients were frail, compared with only 30% of CORO patients and 7% of PCI patients. These data confirm that the more severe CVD patients (HF and CABG) more often suffer from (more severe) frailty. Indeed, while moderate frailty was mostly detected in CABG and HF patients, CORO and PCI patients mostly suffered from minor frailty. These higher prevalence rates and more severe levels of frailty in HF patients could be mainly explained by the more severe disease characteristics, such as dyspnoea, exhaustion or peripheral oedema. This is further confirmed by the high prevalence rates of frailty in older (≥80 years) as well as in younger (<80 years) HF patients (82.4% vs. 53.8% according to Vigorito and 85.3% vs. 50.0% according to Fried). However, severe frailty was not detected even in the most severe CVDs such as HF. This could be explained by the fact that most HF patients were classified as New York Heart Association (NYHA) class II or III. Frailty was more prevalent in females than in males in the total population (53% vs. 38%) as well as within each CVD individually. This was mainly due to significantly lower/worse outcomes in gait speed, handgrip strength and TUG and a trend for a lower MNA score, although lower results can be expected in females than in males. We thus can conclude that, despite the finding that most of the participants were not frail or mildly frail, CABG and HF patients are especially at risk for developing or experiencing frailty, particularly females, which is supported by previous evidence [12]. Frailty is related to several adverse health outcomes, such as functional decline with an increased risk of dependency (because of falls, difficulties with mobility, impairment of basic and instrumental ADL), poor cognition (with an increased incidence of dementia and delirium) and a decreased quality of life (subjective health, mood, engagement and social relations), resulting in increased healthcare consumption with more frequent hospitalisations (such as emergency room visits and surgical complications), institutionalisation and, finally, premature death [11]. Therefore, it might be advisable to execute frailty screenings more often in clinical practice in these patients and initiate preventive measures accordingly. In this regard, exercise training, in combination with nutritional support, is highly recommended [37,38,39,40,41]. 

Along with the potential of the Vigorito frailty assessment tool to detect frailty in several domains (physical, psychosocial, cognitive) in CVD patients (in contrast to the Fried phenotype), Vigorito et al.’s frailty assessment tool reported a lower prevalence of frailty (44%) and of minor (34%) compared to moderate (10%) frailty. The Fried phenotype reported a larger percentage of frailty (64%) and of frail compared to pre-frail patients. By examining frailty in several domains, Vigorito et al.’s tool has the capacity to only consider a patient frail when several domains are affected and could be more sensitive in detecting small differences in frailty severity, while the Fried tool may have a smaller latitude and be limited by a ceiling effect. Moreover, the Fried phenotype can be somewhat subjective, as, for example, the two questions regarding exhaustion are often difficult for patients to answer correctly. Furthermore, registration of involuntary weight loss only does not always fully capture the nutritional status of the patients. 

However, the Vigorito frailty assessment tool is not yet validated in CVD patients. Therefore, based on all frailty measurements that we performed in this study, we tried to analyse which measurements could contribute to the prediction of frailty and related hospitalisations and mortality in CVD patients based on the model proposed by Vigorito et al. Based on multivariate regression analysis, sex, MNA, Katz scale, TUG, handgrip strength, MMSE, GDS-15 and total number of medications are collectively the best predictors of frailty (model R^2^ = 0.95). Based on this specific frailty assessment tool, which comprises components of Vigorito et al.’s frailty assessment tool, the presence of frailty in CVD patients can be feasibly detected in a time- and cost-efficient way, as is it takes only 10–15 min, while, except for a handgrip dynamometer, no expensive equipment is required. Thus, this score calculator can be implemented in clinical practice and/or validated in subsequent studies:

[(18.221173 + (1.1454217 × sex] + (−0.267283 × MNA score)] + (−0.947011 × Katz scale score) + (0.2157993 × TUG score) + (−0.081659 × handgrip strength score) + [−0.18281 × MMSE score) + (0.2700342 × GDS-15 score) + (0.2264091 × total number of medications) + [0.0453303 × (Katz scale score − 5.21805) × (TUG score − 14.3608)]]

Moreover, given the importance of avoiding false-negative frailty diagnoses in clinical practice, a cut-off score corresponding to a sensitivity of 1.00 was determined. According to this model, a frailty diagnosis is thus made with a score of 5.56 or higher, which corresponds to a 100% probability of correctly detecting frailty with a false-positive probability of 46%. 

Finally, we examined whether frailty is related to 6-month clinical outcomes. As all three frailty assessment batteries (Fried χ^2^ = 10.431, *p* = 0.002; Vigorito χ^2^ = 7.755, *p* = 0.011; and the newly developed battery χ^2^ = 5.953, *p* = 0.017) found significant associations between frailty and mortality, we can conclude that frailty indeed increases the mortality risk. These increased mortality rates in frail CVD patients were previously confirmed in a recent systematic review [12]. Moreover, given the significant association between hospitalisations and frailty according to Fried, there are indications that frailty also increases the risk for (urgent) hospital admissions. Based on a logistic regression model, the stronger association of frailty with mortality, in comparison with hospitalisations, was further confirmed, given the significant associations between several frailty assessment components (MNA, Katz scale, walking time, gait speed, TUG, MMSE and number of medications) and mortality in comparison with hospitalisations (only walking time, handgrip strength and GDS). It thus seems possible that frailty in CVD patients is more related to increased mortality instead of increased risk for hospitalisation. Moreover, when we examine the specific frailty components of Fried vs. Vigorito, it seems that mainly specific Vigorito components are more able to predict mortality, while specific Fried components can better predict 6-month (urgent) hospitalisations. Furthermore, as especially orthopaedic hospital admissions were associated with frailty and several frailty components, there are indications that especially a low handgrip strength and gait speed, a worse nutritional status and a depressed state can result in hospital admissions due to fall incidents and related fractures. These findings again confirm the importance of the early detection and multidisciplinary treatment of frailty in order to prevent hospitalisations and mortality. 

Based on the multivariate regression model, we were able to select specific frailty measurements that were highly qualified to predict frailty. Based on this newly proposed frailty assessment tool, it will now be possible to examine frailty in a sex-specific and multidimensional way. Moreover, by using the proposed formula, the exact score of each frailty measurement can be input, which will then result in an automatic and therefore simple and time-efficient calculation of the frailty score. As this easy-to-use tool does not necessitate extensive education, it will therefore be accessible for all members of the healthcare professional, which will further encourage a multidisciplinary frailty approach. Usage of this exact score is an important advantage over the Vigorito tool, in which it is unclear how raw data of MNA and TUG should be rounded to interpret the frailty severity. Moreover, the Vigorito tool only takes into account specific criteria for men vs. women for the handgrip strength criteria, in contrast to other sex-influenced criteria such as TUG and gait speed. Furthermore, based on the sensitivity and specificity curves (Table 5), it will be possible to check the sensitivity and accompanying specificity of the preferred cut-off scores. We thus can conclude that this newly developed frailty assessment battery provides several advantages over the Fried and Vigorito tools to more objectively examine frailty in CVD patients. 

Some study limitations should be taken into account. First, the sample sizes were not equal across all of the different CVDs, and especially CABG patients were underrepresented in this study. Second, the frailty assessment battery was not performed on the same day of hospitalisation for all patients, which could have caused differences in the physical status of the patients. Moreover, a modified version of the Fried criteria was used by implementing the Katz scale to examine the level of physical activity instead of the original Minnesota Leisure Time Activity questionnaire. Although this Katz scale was more in accordance with the study population, the use of a modified version of the Fried criteria has to be acknowledged. Moreover, as no severely frail patients were detected in this study, it may be worthwhile to further evaluate the diagnostic power of the Vigorito frailty assessment tool in a larger population of CVD patients. Furthermore, we aimed to develop a new frailty assessment battery based on a multivariate regression model with the total Vigorito frailty score as a dependent variable. However, it remains important to acknowledge that this Vigorito frailty assessment tool has not yet been validated and thus requires further research. 

Finally, there are indications that certain biomarkers (such as NT-proBNP) may be associated with the presence of frailty in older HF patients. To further optimize frailty diagnosis, it may thus be promising to explore the potential role of biomarkers in future research [42].

## 5. Conclusions

To detect frailty, including at an early stage, in patients with CVD, sex, MNA, Katz scale, TUG, handgrip strength, MMSE, GDS-15 and total number of medications play a key role. A new simple, time- and cost-efficient test battery for frailty with sufficient sensitivity and specificity, accessible for all healthcare professionals, is proposed in this study.

## Figures and Tables

**Table 1:** Baseline characteristics of the study population according to sex and CVD.

		Total	CORO	PCI	CABG	HF
*n* (%)	Total	133	27 (20.3)	30 (22.6)	16 (12.0)	60 (45.1)
	M	76 (57.1)	14 (51.9)	19 (63.3)	14 (87.5) †	29 (48.3)
	F	57 (42.9)	13 (48.1)	11 (36.7)	2 (12.5)	31 (51.7)
Age (Years)	Total	78.1 ± 6.7	75.4 ± 5.3 *	75.5 ± 6.5 **	77.0 ± 7.6	80.9 ± 6.1
	M	77.2 ± 6.9	74.0 ± 4.1	75.9 ± 7.1	76.4 ± 7.9	79.9 ± 6.6
	F	79.4 ± 6.3	77.0 ± 6.1	74.8 ± 5.6	80.8 ± 5.1	81.9 ± 5.5
Body length (cm)	Total	166.3 ± 9.7	167.3 ± 10.1	166.9 ± 9.8	169.8 ± 6.5	164.7 ± 10.0
	M	172.4 ± 6.4 †	175.0 ± 6.3 †	172.3 ± 5.8 †	171.2 ± 5.5 †	171.8 ± 7.2 †
	F	158.2 ± 7.0	158.9 ± 5.6	157.7 ± 8.3	159.5 ± 0.7	158.0 ± 7.4
Body weight (kg)	Total	74.0 ± 13.4	78.2 ± 14.5	74.9 ± 12.1	76.0 ± 13.2	71.2 ± 13.2
	M	78.4 ± 12.1 †	82.3 ± 13.0	79.2 ± 12.2 †	77.4 ± 13.4	76.4 ± 11.0 †
	F	68.3 ± 12.8	73.8 ± 15.2	67.3 ± 7.5	66.1 ± 4.8	66.4 ± 13.3
BMI (kg/m^2^)	Total	26.7 ± 4.2	27.9 ± 4.4	26.8 ± 3.2	26.3 ± 3.8	26.3 ± 4.6
	M	26.4 ± 3.6	26.9 ± 3.9	26.7 ± 3.5	26.4 ± 4.0	25.9 ± 3.3
	F	27.3 ± 4.9	29.1 ± 4.8	27.1 ± 2.8	26.0 ± 2.1	26.7 ± 5.5
Overweight % prevalence	Total	67 (50.4)	16 (59.3)	19 (63.3)	6 (37.5)	26 (43.3)
	M	40 (30.1)	8 (29.6)	12 (40.0)	5 (31.3)	15 (25.0)
	F	27 (20.3)	8 (29.6)	7 (23.3)	1 (6.3)	11 (18.3)
Obesity % prevalence	Total	22 (16.5)	6 (22.2)	4 (13.3)	2 (12.5)	10 (16.7)
	M	8 (6.0)	2 (7.4)	2 (6.7)	2 (12.5)	2 (3.3)
	F	14 (10.5)	4 (14.8)	2 (6.7)	0 (0.0)	8 (13.3)
Hypertension % prevalence	Total	120 (90.2)	21 (77.8)	23 (76.7)	16 (100)	60 (100)
	M	66 (49.6)	9 (33.3)	14 (46.7)	14 (87.5)	29 (48.3)
	F	54 (40.6)	12 (44.4)	9 (30.0)	2 (12.5)	31 (51.7)
Type 2 diabetes % prevalence	Total	36 (27.1)	6 (22.2)	5 (16.7)	3 (18.8)	22 (36.7)
	M	20 (15.0)	4 (14.8)	4 (13.3)	3 (18.8)	9 (15.0)
	F	16 (12.0)	2 (7.4)	1 (3.3)	0 (0.0)	13 (21.7)
Dyslipidaemia % prevalence	Total	102 (76.7)	19 (70.4)	27 (90.0)	15 (93.8)	41 (68.3)
	M	61 (45.9)	11 (40.7)	17 (56.7)	13 (81.3)	20 (33.3)
	F	41 (30.8)	8 (29.6)	10 (33.3)	2 (12.5)	21 (35.0)
NYHA	Total					
Class I–II		-	-	-	-	1 (1.7)
Class II		-	-	-	-	13 (21.7)
Class II–III		-	-	-	-	16 (26.7)
Class III		-	-	-	-	17 (28.3)
Class III–IV		-	-	-	-	3 (5.0)
Class IV		-	-	-	-	2 (3.3)
Unknown		-	-	-	-	8 (13.3)
		Total	CORO	PCI	CABG	HF
Cardioprotective medication						
Beta blockers		89 (66.9)	14 (51.9)	19 (63.3)	14 (87.5)	42 (70.0)
Calcium antagonists		37 (27.8)	10 (37.0)	5 (16.7)	3 (18.8)	19 (31.7)
ACE inhibitors		44 (33.1)	4 (14.8)	12 (40.0)	7 (43.8)	21 (35.0)
Angiotensin II receptor blockers		25 (18.8)	6 (22.2)	5 (16.7)	2 (12.5)	12 (20.0)
Diuretics		78 (58.6)	5 (18.5)	10 (33.3)	10 (62.5)	53 (88.3)
Amiodarone		30 (22.6)	0 (0.0)	3 (10.0)	0 (0.0)	27 (45.0)
Sotalol		2 (1.5)	2 (7.4)	0 (0.0)	0 (0.0)	0 (0.0)
Flecainide		4 (3.0)	1 (3.7)	0 (0.0)	0 (0.0)	3 (5.0)
Anticoagulants		123 (92.5)	26 (96.3)	30 (100)	14 (87.5)	53 (88.3)
Ezetimibe		8 (6.0)	0 (0.0)	2 (6.7)	2 (12.5)	4 (6.7)
Statins		101 (75.9)	19 (70.4)	26 (86.7)	15 (93.8)	41 (68.3)
Nitrates		16 (12.0)	6 (22.2)	1 (3.3)	2 (12.5)	7 (11.7)
Sacubitril/Valsartan		4 (3.0)	0 (0.0)	0 (0.0)	0 (0.0)	4 (6.7)
Ivabradine		1 (0.8)	0 (0.0)	0 (0.0)	0 (0.0)	1 (1.7)
Molsidomine		12 (9.0)	4 (14.8)	3 (10.0)	4 (25.0)	1 (1.7)
Metformin		23 (17.3)	4 (14.8)	4 (13.3)	3 (18.8)	12 (20.0)
Sulphonylurea		4 (3.0)	1 (3.7)	1 (3.3)	1 (6.3)	1 (1.7)
Glinides/meglitinides		4 (3.0)	0 (0.0)	2 (6.7)	0 (0.0)	2 (3.3)
GLP1 analogues		1 (0.8)	0 (0.0)	0 (0.0)	0 (0.0)	1 (1.7)
DPP4 inhibitors		5 (3.8)	0 (0.0)	1 (3.3)	0 (0.0)	4 (6.7)
SGLT2 inhibitors		4 (3.0)	1 (3.7)	1 (3.3)	0 (0.0)	2 (3.3)
Insulin (ultrafast-acting)		3 (2.3)	1 (3.7)	0 (0.0)	0 (0.0)	2 (3.3)
Insulin (fast-acting)		2 (1.5)	0 (0.0)	0 (0.0)	0 (0.0)	2 (3.3)
Insulin (intermediate)		1 (0.8)	0 (0.0)	0 (0.0)	0 (0.0)	1 (1.7)
Insulin (slow-acting)		7 (5.3)	2 (7.4)	0 (0.0)	0 (0.0)	5 (8.3)
Opioids		10 (7.5)	2 (7.4)	0 (0.0)	0 (0.0)	8 (13.3)
Analgesics		29 (21.8)	3 (11.1)	0 (0.0)	8 (50.0)	18 (30.0)

BMI, body mass index; CABG, coronary artery bypass grafting; cm, centimetre; CORO, coronarography; CVD, cardiovascular disease; HF, heart failure; kg, kilogram; m, metre; *n*, number; NYHA, New York Heart Association; PCI, percutaneous coronary intervention; SD, standard deviation; *p* < 0.05 * CORO vs. HF; ** PCI vs. HF; † *p* < 0.05 between sexes. Results are expressed as mean ± SD or as *n* (% within CVD group) (for results per CVD) or as *n* (% within total population) (for results of the total population).

**Table 2:** Number of frail subjects according to CVD and sex and analysis of the frailty component scores based on the Fried frailty assessment tool.

			Total (*n* = 133)	CORO (*n* = 27) M (*n* = 14) F (*n* = 13)	PCI (*n* = 30) M (*n* = 19) F (*n* = 11)	CABG (*n* = 16) M (*n* = 14) F (*n* = 2)	HF (*n* = 60) M (*n* = 29) F (*n* = 31)
Weight loss	Total		20	1	3	4	12
	M	Frail *n* (%)	13 (17.1)	1 (7.1)	3 (15.8)	4 (28.6)	5 (17.2)
	F		7 (12.3)	0 (0.0)	0 (0.0)	0 (0.0)	7 (22.6)
Exhaustion							
I felt that everything I did was an effort	Total	Raw score	1.4 ± 1.2	1.1 ± 1.1 *	0.4 ± 0.7 **	1.3 ± 1.3	2.0 ± 1.1
	M		1.0 ± 1.1 †	0.7 ± 0.9	0.2 ± 0.5	1.2 ± 1.4	1.6 ± 1.1 †
	F		1.8 ± 1.1	1.5 ± 1.1	0.7 ± 0.8	1.5 ± 0.7	2.4 ± 0.9
I could not get going	Total	Raw score	1.4 ± 1.3	0.8 ± 1.1 *	0.6 ± 0.9 *	1.6 ± 1.4	2.0 ± 1.2
	M		1.1 ± 1.2 †	0.6 ± 0.9	0.5 ± 0.8	1.5 ± 1.4	1.6 ± 1.2 †
	F		1.7 ± 1.3	1.0 ± 1.3	0.8 ± 1.1	2.0 ± 1.4	2.4 ± 1.0
Total	M	Frail *n* (%)	21 (27.6)	1 (7.1)	1 (5.3)	5 (35.7)	14 (48.3)
	F		30 (52.6)	4 (30.8)	1 (9.1)	0 (0.0)	25 (80.6)
Gait speed (m/s)	Total	Raw score	0.87 ± 0.48	1.03 ± 0.44 *	1.27 ± 0.36 **	0.92 ± 0.48 ***	0.59 ± 0.36
	M	Raw score	0.98 ± 0.52 †	1.21 ± 0.47 †	1.34 ± 0.40	0.98 ± 0.48	0.63 ± 0.40
		Frail *n* (%)	27 (35.5)	2 (14.3)	0 (0.0)	5 (35.7)	20 (69.0)
	F	Raw score	0.73 ± 0.38	0.85 ± 0.31	1.15 ± 0.24	0.51 ± 0.24	0.55 ± 0.33
		Frail *n* (%)	33 (57.9)	6 (46.2)	1 (9.1)	1 (50.0)	25 (80.6)
Level of physical activity (Katz independence in ADL)	Total	Raw score	5.2 ± 1.3	5.5 ± 1.1 *	6.0 ± 0.0 **	5.4 ± 1.1	4.7 ± 1.5
	M	Raw score	5.3 ± 1.3	5.6 ± 1.1	6.0 ± 0.0	5.3 ± 1.1	4.7 ± 1.5
		Frail *n* (%)	24 (31.6)	2 (14.3)	0 (0.0)	5 (35.7)	17 (58.6)
	F	Raw score	5.1 ± 1.3	5.5 ± 1.1	6.0 ± 0.0	6.0 ± 0.0	4.6 ± 1.5
		Frail *n* (%)	20 (35.1)	3 (23.1)	0 (0.0)	0 (0.0)	17 (54.8)
Handgrip strength (kg)	Total	Raw score	26.7 ± 11.8	30.7 ± 13.2 *	33.1 ± 11.1 **	31.1 ± 9.0 ***	20.5 ± 9.1
	M	Raw score	33.3 ± 10.7 †	38.8 ± 13.3 †	39.0 ± 9.3 †	33.0 ± 7.7	27.1 ± 7.9 †
		Frail *n* (%)	31 (40.8)	3 (21.4)	5 (26.3)	3 (21.4)	20 (69.0)
	F	Raw score	17.9 ± 6.2	22.0 ± 4.9	22.9 ± 4.7	17.9 ± 6.2	14.4 ± 4.9
		Frail *n* (%)	35 (61.4)	4 (30.8)	3 (27.3)	1 (50.0)	27 (87.1)
Total frailty score	Total	Raw score	1.8 ± 1.6	1.0 ± 1.2 *	0.5 ± 0.8 **	1.5 ± 1.5 ***	3.0 ± 1.4
	M		1.5 ± 1.6 †	0.6 ± 1.2	0.5 ± 0.8	1.6 ± 1.6	2.6 ± 1.4
	F		2.2 ± 1.6	1.3 ± 1.3	0.5 ± 0.7	1.0 ± 1.4	3.3 ± 1.2

ADL, activities of daily living; CABG, coronary artery bypass grafting; CORO, coronarography; F females; HF, heart failure; kg, kilogram; M, males; *n*, number; PCI, percutaneous coronary intervention; s, seconds; SD, standard deviation; *p* < 0.05 * CORO vs. HF; ** PCI vs. HF; *** CABG vs. HF; † *p* < 0.05 between sexes. Results are expressed as mean ± SD.

**Table 3:** Frailty assessment using Vigorito et al.’s tool.

			CORO		PCI		CABG		HF	
			M (*n* = 14)	F (*n* = 13)	M (*n* = 19)	F (*n* = 11)	M (*n* = 14)	F (*n* = 2)	M (*n* = 29)	F (*n* = 31)
MNA	NF/MiF/ModF/SF	*n*	10/3/1/0	9/3/1/0	16/3/0/0	8/2/1/0	7/5/2/0	0/2/0/0	9/15/5/0	4/16/7/4
		%	71.4/21.4/7.1/0.0	69.2/23.1/7.7/0.0	84.2/15.8/0.0/0.0	72.7/18.2/9.1/0.0	50.0/35.7/14.3	0.0/100/0.0/0.0	31.0/51.7/17.2/0.0	12.9/51.6/22.6/12.9
Katz independence in ADL	NF/MiF/ModF/SF	*n*	12/2/0/0	10/3/0/0	19/0/0/0	11/0/0/0	11/3/0/0	2/0/0/0	19/6/4/0	17/12/2/0
		%	85.7/14.3/0.0/0.0	76.9/23.1/0.0/0.0	100/0.0/0.0/0.0	100/0.0/0.0	78.6/21.4/0.0/0.0	100/0.0/0.0/0.0	65.5/20.7/13.8/0.0	54.8/38.7/6.5/0.0
Gait speed	NF/MiF/ModF/SF	*n*	11/2/0/1	6/3/4/0	17/2/0/0	10/1/0/0	8/1/4/1	0/1/0/1	7/5/7/10	5/2/14/10
		%	78.6/14.3/0.0/7.1	46.2/23.1/30.8/0.0	89.5/10.5/0.0/0.0	90.9/9.1/0.0/0.0	57.1/7.1/28.6/7.1 *	0.0/50.0/0.0/50.0	24.1/17.2/24.1/34.5	16.1/6.5/45.2/32.3
TUG	NF/MiF/ModF/SF	*n*	11/2/0/1	6/2/4/1	17/2/0/0	8/2/1/0	8/2/3/1	1/0/0/1	6/5/8/10	6/6/10/9
		%	78.6/14.3/0.0/7.1	46.2/15.4/30.8/7.7	89.5/10.5/0.0/0.0	72.7/18.2/9.1/0.0	57.1/14.3/21.4/7.1	50.0/0.0/0.0/50.0	20.7/17.2/27.6/34.5	19.4/19.4/32.3/29.0
Handgrip strength	NF/MiF/ModF/SF	*n*	11/1/1/1	13/0/0/0	14/3/2/0	10/1/0/0	10/2/1/1	1/1/0/0	9/8/10/2	14/7/9/1
		%	78.6/7.1/7.1/71	100/0.0/0.0/0.0	73.7/15.8/10.5/0.0	90.9/9.1/0.0/0.0	71.4/14.3/7.1/7.1	50.0/50.0/0.0/0.0	31.0/27.6/34.5/6.9	45.2/22.6/29.0/3.2
MMSE	NF/MiF/ModF/SF	*n*	13/1/0/0	10/2/1/0	19/0/0/0	11/0/0/0	13/0/0/1	2/0/0/0	16/11/2/0	22/5/3/1
		%	92.9/7.1/0.0/0.0	76.9/15.4/7.7/0.0	100/0.0/0.0/0.0	100/0.0/0.0/0.0	92.9/0.0/0.0/7.1	100/0.0/0.0/0.0	55.2/37.9/6.9/0.0	71.0/16.1/9.7/3.2
GDS	NF/MiF/ModF/SF	*n*	7/5/2/0	8/2/3/0	14/4/1/0	6/4/1/0	8/4/2/0	0/2/0/0	8/16/5/0	9/15/7/0
		%	50.0/35.7/14.3/0.0	61.5/15.4/23.1/0.0	73.7/21.1/5.3/0.0	54.5/36.4/9.1/0.0	57.1/28.6/14.3/0.0	0.0/100/0.0/0.0	27.6/55.2/17.2/0.0	29.0/48.4/22.6/0.0
Number of medications	NF/MiF/ModF/SF	*n*	5/6/3/0	4/5/3/1	3/11/5/0	2/4/5/0	0/8/5/1	0/1/1/0	1/12/8/8	0/16/7/8
		%	35.7/42.9/21.4/0.0	30.8/38.5/23.1/7.7	15.8/57.9/26.3/0.0	18.2/36.4/45.5/0.0	0.0/57.1/35.7/7.1	0.0/50.0/50.0/0.0	3.4/41.4/27.6/27.6	0.0/51.6/22.6/25.8

CABG, coronary artery bypass grafting; CORO, coronarography; F, females; HF, heart failure; M, males; MiF, mild frailty; ModF, moderate frailty; *n*, number; NF, not frail; PCI, percutaneous coronary intervention; SF, severe frailty; TUG, timed up-and-go test. Results are expressed as *n* or % (% within males or within females per CVD); * p < 0.05 association between level (severity) of frailty and sex per CVD.

**Table 4:** Analysis of the frailty component scores, according to CVD and sex, based on Vigorito et al.’s frailty assessment tool.

		Total (*n* = 133)	CORO (*n* = 27)	PCI (*n* = 30)	CABG (*n* = 16)	HF (*n* = 60)
MNA (/30)	Total	23.6 ± 3.6	25.8 ± 3.2 *	25.3 ± 2.2 **	23.8 ± 3.2	21.8 ± 3.4
	M	24.2 ± 3.1	25.8 ± 3.3	25.5 ± 1.7	23.8 ± 3.4	22.9 ± 2.9 †
	F	22.8 ± 4.0	25.8 ± 3.2	25.0 ± 2.9	23.8 ± 0.4	20.8 ± 3.7
Katz independence in ADL (*n*)	Total	5.2 ± 1.3	5.5 ± 1.1 *	6.0 ± 0.0 **	5.4 ± 1.1	4.7 ± 1.5
	M	5.3 ± 1.3	5.6 ± 1.1	6.0 ± 0.0	5.3 ± 1.1	4.7 ± 1.5
	F	5.1 ± 1.3	5.5 ± 1.1	6.0 ± 0.0	6.0 ± 0.0	4.6 ± 1.5
Gait speed (m/s)	Total	0.87 ± 0.48	1.03 ± 0.44 *	1.27 ± 0.36 **	0.92 ± 0.48 ***	0.59 ± 0.36
	M	0.98 ± 0.52 †	1.21 ± 0.47 †	1.34 ± 0.40	0.98 ± 0.48	0.63 ± 0.40
	F	0.73 ± 0.38	0.85 ± 0.31	1.15 ± 0.24	0.51 ± 0.24	0.55 ± 0.33
TUG (s)	Total	14.4 ± 9.0	11.5 ± 6.9 *	8.3 ± 2.5 **	12.9 ± 7.7	19.1 ± 9.8
	M	13.5 ± 9.3 †	10.3 ± 8.4 †	7.8 ± 2.1	11.8 ± 6.3	19.5 ± 10.5
	F	15.6 ± 8.5	12.9 ± 4.8	9.2 ± 2.9	20.6 ± 14.9	18.7 ± 9.3
Handgrip strength (kg)	Total	26.7 ± 11.8	30.7 ± 13.2 *	33.1 ± 11.1 **	31.1 ± 9.0 ***	20.5 ± 9.1
	M	33.3 ± 10.7 †	38.8 ± 13.3 †	39.0 ± 9.3 †	33.0 ± 7.7	27.1 ± 7.9 †
	F	17.9 ± 6.2	22.0 ± 4.9	22.9 ± 4.7	17.9 ± 6.2	14.4 ± 4.9
MMSE (/30)	Total	26.2 ± 3.2	27.3 ± 2.5 *	27.6 ± 1.7 **	26.8 ± 4.1 ***	24.9 ± 3.4
	M	26.3 ± 3.3	27.7 ± 2.2	27.7 ± 1.7	26.6 ± 4.3	24.6 ± 3.3
	F	26.1 ± 3.2	26.9 ± 2.9	27.5 ± 1.8	28.0 ± 1.4	25.1 ± 3.4
GDS-15 (/15)	Total	3.2 ± 2.3	3.3 ± 3.0	2.2 ± 1.8 **	2.8 ± 1.9	3.9 ± 2.0
	M	3.0 ± 2.2	3.3 ± 2.7	2.0 ± 1.6	2.5 ± 2.0	3.9 ± 2.1
	F	3.5 ± 2.4	3.4 ± 3.4	2.6 ± 2.0	4.5 ± 0.7	3.8 ± 2.0
Number of medications (*n*)	Total	8.3 ± 3.4	6.6 ± 3.2 *	7.2 ± 2.4 **	7.8 ± 2.5	9.9 ± 3.4
	M	8.2 ± 3.6	6.2 ± 3.4	6.8 ± 2.3	7.8 ± 2.6	10.0 ± 3.8
	F	8.6 ± 3.1	6.9 ± 3.0	7.7 ± 2.4	7.5 ± 2.1	9.7 ± 3.2
Total frailty score	Total	6.2 ± 4.8	3.8 ± 3.8 *	2.4 ± 2.1 **	5.6 ± 4.2	9.4 ± 4.2
	M	5.6 ± 4.7	3.2 ± 3.7	2.2 ± 2.0	5.3 ± 4.4	9.2 ± 4.2
	F	7.0 ± 4.8	4.5 ± 4.0	2.7 ± 2.5	7.5 ± 3.5	9.6 ± 4.3

ADL, activities of daily living; CABG, coronary artery bypass grafting; cm, centimetre; CORO, coronarography; F, females; GDS, Geriatric Depression Scale; HF, heart failure; kg, kilogram; M, males; m, metre; MNA, Mini Nutritional Assessment; MMSE, Mini-Mental State Examination; *n*, number; PCI, percutaneous coronary intervention; s, seconds; SD, standard deviation; TUG, timed up-and-go test; *p* < 0.05 * CORO vs. HF; ** PCI vs. HF; *** CABG vs. HF; † *p* < 0.05 between sexes. Results are expressed as mean ± SD.

**Table 5:** Cut-off scores and corresponding sensitivity and specificity analyses of the newly developed frailty assessment battery.

Cut-Off Score	Sensitivity	Specificity
−1.71	1.00	0.00
−0.34	1.00	0.03
0.09	1.00	0.06
0.42	1.00	0.08
0.65	1.00	0.10
0.85	1.00	0.13
1.09	1.00	0.17
3.04	1.00	0.35
**5.56**	**1.00**	**0.54**
7.02	0.63	0.60
7.17	0.50	0.60
7.27	0.38	0.60
7.46	0.25	0.61
7.92	0.13	0.64
9.09	0.00	0.67
11.2	0.00	0.83
13.3	0.00	0.91
15.07	0.00	0.96
17.32	0.00	0.98
18.90	0.00	1.00

Note: The bold format indicates the preferred cut-off score which should be used when implementing the newly proposed frailty assessment tool to detect frailty.

**Table 6:** Significant associations between markers of frailty and 6-month clinical outcomes.

6-Month Clinical Outcomes	Frailty Marker	*p*-Value
Mortality	Frailty status according to Fried	*p* = 0.002
	Frailty status according to Vigorito	*p* = 0.011
	MNA	*p* = 0.003
	Gait speed	*p* = 0.023
	TUG	*p* = 0.001
	MMSE	*p* = 0.042
	Handgrip strength	*p* = 0.006
	Frailty status according to the newly developed frailty assessment battery	*p* = 0.017
6-month hospitalisations	Frailty status according to Fried	*p* = 0.030
	Handgrip strength	*p* = 0.004
	Exhaustion	*p* = 0.011
6-month urgent hospitalisations	Frailty status according to Fried	*p* = 0.032
	Handgrip strength	*p* = 0.013 (Fried) *p* = 0.019 (Vigorito)
	Exhaustion	*p* = 0.032
	Physical activity	*p* = 0.03
	Frailty status according to the newly developed frailty assessment battery	*p* = 0.04
Orthopaedic hospitalisations	Frailty status according to Fried	*p* = 0.005
	Handgrip strength	*p* = 0.033
	Gait speed	*p* = 0.023
	Frailty status according to Vigorito	*p* = 0.022
	Gait speed	*p* = 0.025
	MNA	*p* = 0.018
	GDS-15	*p* = 0.003
Cardiovascular hospitalisations	Handgrip strength (Fried)	*p* = 0.028

GDS-15, Geriatric Depression Scale; MMSE, Mini-Mental State Examination; MNA, Mini Nutritional Assessment; TUG, timed up-and-go test.

## Data Availability

All data tables are included in this manuscript.

## Appendix A

**Table A1:** Frailty phenotype according to Fried et al. [13].

Weight loss	“In the last year, have you lost more than 10 pounds unintentionally (i.e., not due to dieting or exercise)?” If yes, then frail for weight loss criterion. At follow-up, weight loss was calculated as: (Weight in previous year–current measured weight)/(weight in previous year) = K. If K ≥ 0.05 and the subject does not report that he/she was trying to lose weight (i.e., unintentional weight loss of at least 5% of previous year’s body weight), then frail for weight loss = Yes.	
Exhaustion	Using the CES–D Depression Scale, the following two statements are read. (a) I felt that everything I did was an effort; (b) I could not get going. The question is asked: “How often in the last week did you feel this way?” 0 = rarely or none of the time (<1 day) 1 = some or a little of the time (1–2 days) 2 = a moderate amount of the time (3–4 days) 3 = most of the time. Subjects answering “2” or “3” to either of these questions are categorized as frail for the exhaustion criterion.	
Physical activity	Based on the short version of the Minnesota Leisure Time Activity questionnaire, asking about walking, chores (moderately strenuous), mowing the lawn, raking, gardening, hiking, jogging, biking, exercise cycling, dancing, aerobics, bowling, golf, singles tennis, doubles tennis, racquetball, calisthenics and swimming, kcals per week expended are calculated using standardised algorithm. This variable is stratified by gender. Men: Those with kcals of physical activity per week < 383 are frail. Women: Those with kcals per week < 270 are frail.	
Walk time	Cut-off for time to walk 15 feet criterion for frailty (Stratified by gender and height)	
	Men	
	Height ≤ 173 cm	≥7 s
	Height > 173 cm	≥6 s
	Women	
	Height ≤ 159 cm	≥7 s
	Height > 159 cm	≥6 s
Grip strength	Cut-off for grip strength (kg) criterion for frailty (stratified by gender and BMI quartiles)	
	Men	
	BMI ≤ 24	≤29
	BMI 24.1–26	≤30
	BMI 26.1–28	≤30
	BMI > 28	≤32
	Women	
	BMI ≤ 23	≤17
	BMI 23.1–26	≤17.3
	BMI 26.1–29	≤18
	BMI > 29	≤21

BMI, body mass index; kcals, kilocalories; CES-D, Center of Epidemiologic Studies—depression subscale; kg, kilogram.

**Table A2:** Vigorito et al.’s frailty assessment tool.

	No Frailty		Minor Frailty	Moderate Frailty	Severe Frailty
	Score 0		Score 1	Score 2	Score 3
MNA (/30)	A validated screening and assessment tool to identify persons of 65 years or older who are malnourished or at risk of malnutrition based on 6 screening questions and 12 assessment questions. A lower score indicates a higher risk of malnutrition.				
	≥25		21–24	17–20	<17
Katz independence in ADL (6 activities)	A screening tool to examine the level of (in)dependence in activities of daily living (ADL) (bathing, dressing, transfers, toileting, continence and eating). Complete independence in performing these activities results in a score of 1, while any dependence (from partial to full help required) is scored as 0. This results in a total score from 0 to 6 (i.e., number of independent activities), in which the highest score is associated with complete independence in 6 ADLs.				
	5–6 activities		3–4 activities	1–2 activities	0 activities
Gait speed (m/s)	Evaluation of the gait speed (expressed in metres per second (m/s) based on a 4.6 m walking test (use of walking aids is permitted).				
	≥0.80		0.61–0.79	0.40–0.60	<0.40
TUG (s)	A test that evaluates a combination of mobility, balance and lower-extremity strength. The subject has to stand up from a chair (use of armrests permitted), walk 3 m, return and sit down in the chair again as quickly but safely as possible (use of walking aids is permitted). The walking time is registered in seconds.				
	≤10		11–14	15–20	>20
Handgrip strength (kg)	Evaluation of the handgrip strength (kg) of the dominant hand with a handheld dynamometer. The subject has to squeeze three times, and the highest value is taken into account for the evaluation of frailty severity.				
	F	>15.6	11.4–15.6	7.3–11.3	≤7.2
	M	≥30.6	25.7–30.5	19.0–25.6	≤18.9
MMSE (/30)	A valid and reliable screening tool to detect cognitive disabilities in older adults in the domains of orientation in time and space, registration, attention and calculation, recall, language and copying. A lower score indicates a lower level of cognitive abilities.				
	>24		21–24	16–20	≤15
GDS-15 (/15)	A screening tool for older adults consisting of 15 questions to detect the presence of a depressive mood. A higher score indicates a more depressed state.				
	<3		3–5	6–10	11–15
Number of medications (*n*)	Registration of the use of medications. Vitamins, minerals and food supplements are not included.				
	1–4		5–8	9–12	>12
TOTAL SCORE	0–6		7–12	13–18	19–24

ADL, activities of daily living; GDS. Geriatric Depression Scale; m, metre; MMSE, Mini-Mental State Examination; MNA, Mini Nutritional Assessment; s, seconds; TUG, timed up-and-go test.

**Table A3:** Additional frailty measures.

IPAQ (long version) (METS/min/week)	An evaluation tool that examines the level of physical activity spent in the previous seven days in the domains of work, transportation, domestic/garden and recreation/sport/leisure time as well as the time spent sitting. A higher score indicates a higher level of physical activity.
Muscle strength (kg)	Evaluation of the muscle strength of the knee extensors (sitting position with hip and knee flexed 90°) and hip flexors (supine position with hip flexed 90°) of both legs, measured with the MicroFET^®^ dynamometer (Hoggan Health Industries Inc., West Jordan, UT, USA). Each measurement is repeated three times, and the highest value is used in the data analysis.
Timed chair stand test (s)	A test that evaluates the functional muscle strength of the lower limbs. The subject has to stand up five times from a chair, without using armrests (arms crossed at the chest), and has to return to the sitting position as fast and as safely as possible. The time is registered in seconds.
FES-I (/64)	A questionnaire that examines the level of concern about falling during 16 social and physical activities. A higher score indicates a higher level of concern about falling.

FES-I, Falls Efficacy Scale International; IPAQ, International Physical Activity Questionnaire; kg, kilograms; METS, metabolic equivalents; min, minutes; s, seconds.

**Table A4:** (1) Frailty analysis (in %) according to the Fried phenotype per age group (*p* < 0.001). (2) Frailty analysis (in %) according to the Vigorito frailty assessment tool per age group (*p* = 0.022). (3) Frailty analysis (in %) according to the newly developed frailty assessment tool per age group (*p* < 0.001).

**(1)**		
	**65–75 years**	**>75 years**
Not frail	49.0	26.8
Pre-frail	33.3	22.0
Frail	17.6	51.2
**(2)**		
	**65–75 years**	**>75 years**
Not frail	70.6	46.3
Mild frail	21.6	41.5
Moderate frail	7.8	12.2
**(3)**		
	**65–75 years**	**>75 years**
Not frail	70.6	37.8
Frail	29.4	62.2
